# CORO1A links inflammatory chondrocyte subpopulations to immune microenvironment alterations in osteoarthritis: an integrative multi-omics and single-cell study

**DOI:** 10.3389/fimmu.2026.1820391

**Published:** 2026-06-11

**Authors:** Qiujian Liang, Chao Ning, Shiyao Gao, Dongxin Liu, Jinmin Zhao, Linke Huang, Jun Hou, Zhen Tan

**Affiliations:** 1 Department of Orthopedics, The Second Affiliated Hospital of Guangxi Medical University, Guangxi Medical University, Guangxi, China; 2 Guangxi Key Laboratory of Regenerative Medicine, Guangxi Medical University, Guangxi, China; 3 The First Affiliated Hospital of Guangxi Medical University, Guangxi Medical University, Guangxi, China; 4 Emergency Department of Yunjing Mountain Hospital, Wuhan, China; 5 Emergency Department of Jiangxia District First People’s Hospital, Wuhan, China; 6 Guangxi Diabetic Foot Salvage Engineering Research Center, Guangxi Medical University, Nanning, China

**Keywords:** bioinformatics analysis, chondrocytes, CORO1A, immune microenvironment, osteoarthritis, therapeutic targets

## Abstract

**Aim:**

Osteoarthritis (OA) is increasingly understood as a condition influenced by the diversity of chondrocytes and immune system dysfunction; however, the specific molecular elements connecting distinct chondrocyte populations to the immune environment remain inadequately explored. The protein CORO1A, associated with the cytoskeleton and implicated in immune regulation, has not been thoroughly investigated in the context of OA or through single-cell analysis.

**Approach:**

We employed multi-omics techniques alongside single-cell RNA sequencing, supported by experimental validation using human OA cartilage samples, a murine model of medial meniscus destabilization (DMM), and *in vitro* inflammatory chondrocyte models to elucidate the expression patterns and functional significance of CORO1A in OA.

**Findings:**

The CORO1A gene is recognized as significantly expressed in osteoarthritic cartilage. Transcriptomic evaluations reveal that CORO1A is predominantly expressed in inflammatory and proliferative chondrocyte subpopulations, while showing lower levels in other chondrocyte types, underscoring the specificity of these groups. The upregulation of CORO1A is associated with increased infiltration of various immune cells, including M2 macrophages, plasma cells, and natural killer cells, and is linked to the activation of IL-6/JAK-STAT3 and TNF-α/NF-κB signaling pathways. Additionally, experimental data indicate that CORO1A is markedly elevated in the cartilage regions affected by osteoarthritis in both humans and mice. In terms of its function, the expression of CORO1A in primary chondrocytes is enhanced by inflammatory stimuli, while its suppression leads to reduced MMP13 expression and diminished chondrocyte migration. Conclusion: This study identifies CORO1A as a regulator specific to subpopulations within the immune microenvironment, connecting immune remodeling to both inflammatory and proliferative chondrocytes in OA. Considering the diversity of chondrocytes, the presence of immune cells, and catabolic processes, our results emphasize the significance of CORO1A as a mechanistic factor contributing to cartilage degradation, suggesting its potential as a therapeutic target for OA.

## 1 Introduction

Osteoarthritis (OA) is a long-lasting and degenerative joint condition marked by the gradual deterioration of articular cartilage (1). This ailment primarily affects the body’s weight-bearing joints, such as the knees and hips. As the condition advances, individuals experience significant pain, functional limitations, and reduced mobility, placing a considerable strain on global healthcare systems (2, 3). Knee osteoarthritis (KOA), the most common form of OA, is particularly alarming due to its status as a leading cause of disability worldwide (4). Despite its high prevalence and considerable socioeconomic impact, existing treatment methods largely offer only symptom relief. The main aim of these approaches is to alleviate symptoms and slow disease progression rather than address the underlying structural or functional joint issues (5–7). There is a significant gap in understanding the molecular mechanisms that contribute to the onset and advancement of osteoarthritis. Therefore, it is crucial to investigate the underlying factors involved, which could pave the way for the development of innovative and effective treatment options that go beyond mere symptom management.

The progression of osteoarthritis (OA) is influenced not only by cartilage deterioration but also by complex interactions among various chondrocyte populations and the immune microenvironment (8). Recent studies indicate that communication between immune cells—such as macrophages, T cells, and natural killer (NK) cells—and cartilage plays a significant role in both cartilage damage and repair, rather than merely participating in inflammatory responses (9, 10). Recent studies indicate that communication between immune cells—such as macrophages, T cells, and natural killer (NK) cells—and cartilage plays a significant role in both cartilage damage and repair, rather than merely participating in inflammatory responses. These immune cells are crucial in regulating the remodeling of the extracellular matrix, which involves matrix metalloproteinases (MMPs); their activity must be carefully controlled to maintain a balance between cartilage degradation and regeneration (11). Importantly, M2 macrophages release anti-inflammatory cytokines and growth factors like IGF1 and PDGF, which are also activated during the adaptive immune response in the context of OA (12, 13). Nevertheless, despite growing awareness of chondrocyte diversity and the immune microenvironment’s role in OA, the specific regulatory molecules that govern immune cell polarization related to cartilage repair have yet to be identified.

CORO1A is a crucial protein within the cytoskeleton and belongs to the Coronin protein family, which plays significant roles in various functions, including the movement of immune cells, development of the cytoskeleton, and signaling pathways. These processes are vital for maintaining immune readiness and initiating appropriate inflammatory responses, as highlighted in recent studies (14, 15). In addition to its fundamental cellular roles, CORO1A is increasingly recognized as a pivotal regulator of inflammation and tissue remodeling driven by immune activity. A notable aspect of CORO1A’s function is its dependence on β2 integrin for the adhesion, spreading, and migration of neutrophils under flow conditions, underscoring its essential role in the early stages of inflammatory responses (16).

Additionally, CORO1A has emerged as a key immune-related regulator in inflammatory joint disorders, with gene ontology (GO) terms and Kyoto Encyclopedia of Genes and Genomes (KEGG) pathways associated with CORO1A showing significant enrichment in inflammation and immune signaling. This highlights its potential involvement in the dynamic remodeling of the pathological immune microenvironment (17). Beyond its inflammatory role, CORO1A has been recognized as a modulator of bone resorption, influencing lysosomal secretion and protease release in osteoclasts. This suggests a crucial link between immune cell activity and the mechanisms of bone remodeling (18). Furthermore, alterations in CORO1A expression have been observed in the early stages of osteoarthritis (OA) within both subchondral bone and synovial fluid. These changes are closely associated with inflammatory activity and disease progression, reinforcing the significance of CORO1A in the pathophysiology of OA (19–21).

A key area that warrants further investigation is the role of CORO1A in regulating immune cell activity within the osteoarthritis (OA) immune microenvironment, as well as its specific contributions to cartilage repair mechanisms and disease progression. To comprehensively explore this topic, we undertake a thorough examination of CORO1A’s involvement in OA, combining bioinformatics approaches with experimental validation. Initially, we pinpoint immune-related genes that show differential expression in OA, elucidating their relationship with CORO1A and significant immune-regulatory pathways through Gene Ontology (GO) and Kyoto Encyclopedia of Genes and Genomes (KEGG) enrichment analyses. Subsequently, we identify the immune cell populations most closely associated with CORO1A expression via immune infiltration analyses, with particular emphasis on macrophage polarization, the activity of plasma cells, and the role of natural killer (NK) cells in relation to this protein. Ultimately, we investigate the potential functional roles of CORO1A in altering the immune microenvironment of OA and its implications for cartilage repair processes. Collectively, our findings position CORO1A as a previously overlooked immunoregulatory element in the OA context, offering new insights into its mechanisms and paving the way for potential clinical strategies to modulate immune responses.

## 2 Results

### 2.1 Osteoarthritic cartilage exhibits a distinct and coordinated transcriptional signature

**Figure 1:**
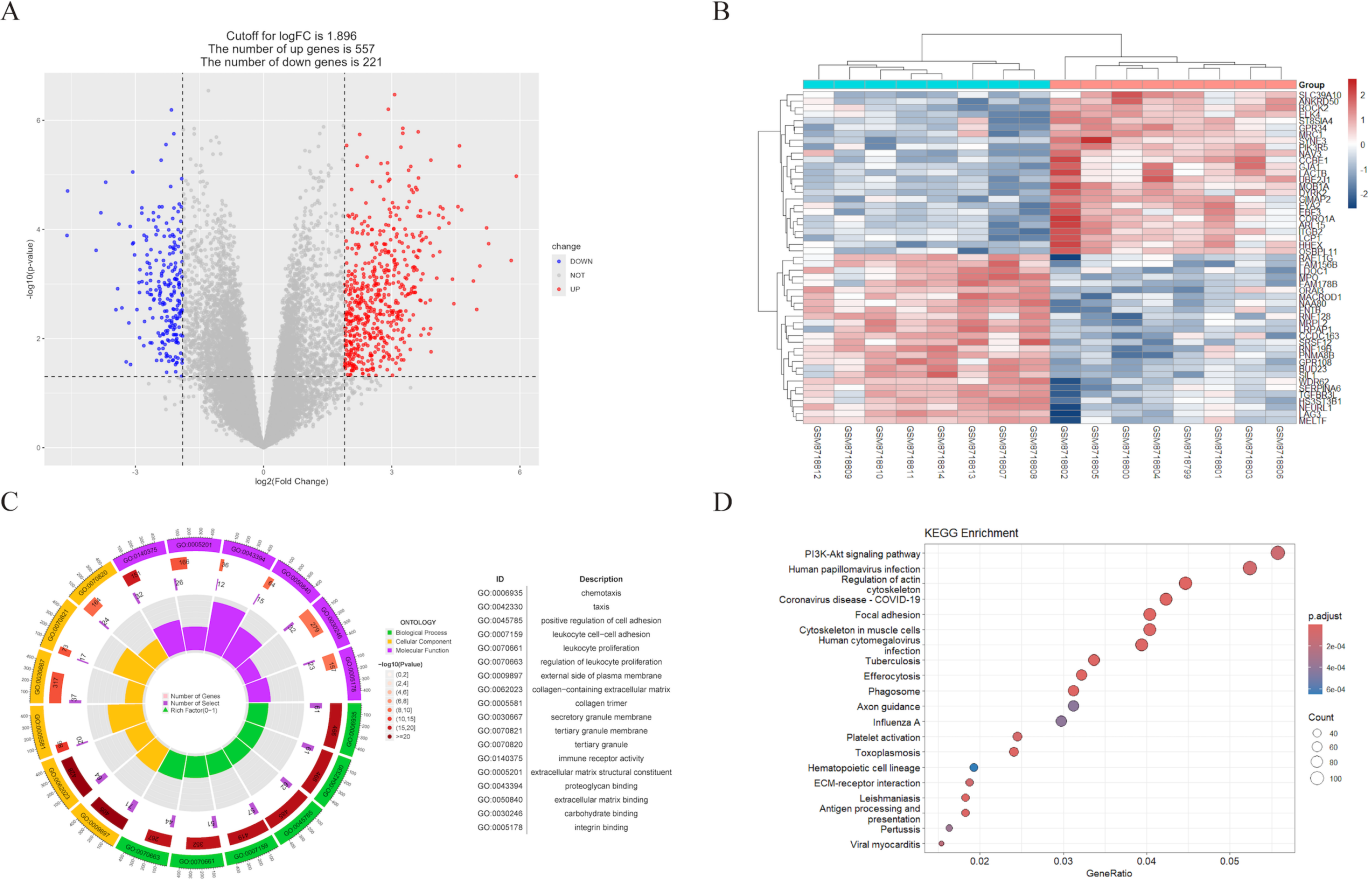
Differential expression analysis reveals distinct transcriptional profiles. **(A)** A volcano graph showed that 778 DEGs were identified between the samples of osteoarthritis (OA) and control groups. The genes that were upregulated were highlighted in red and those that were downregulated were highlighted in blue. **(B)** The clusters were shown as a heat map which was used to represent the expression level of the top 50 and the bottom 50 differentially expressed genes (DEGs). **(C)** GO enrichment analysis of the 621 GO-annotated DEGs among the 778 identified DEGs showing the top ten enriched terms in biological process (BP), cellular component (CC), and molecular function (MF) categories. **(D)** KEGG enrichment analysis of the 778 significant genes identified the signaling pathways that were related to these important genes.

To investigate the relationship between osteoarthritis (OA) and the overall changes in gene expression, rather than focusing solely on individual transcriptional variations, we analyzed the cartilage transcriptomes from the GSE286154 dataset, comparing OA samples with control samples. Our differential expression analysis revealed 778 genes with significant dysregulation, comprising 557 that were upregulated and 221 that were downregulated. This pattern indicates a broad activation of transcription in cartilage affected by OA (**Figure 1A**). Additionally, the relative expression levels of the top 50 upregulated and 50 downregulated DEGs across the OA and control samples were visualized using a heatmap (**Figure 1B**). These findings underscore the unique and reproducible molecular characteristics of OA cartilage. Collectively, these results imply that OA is marked by a consistent reorganization of transcription, laying a strong foundation for further functional annotation and network-level studies aimed at identifying key regulators associated with the condition.

### 2.2 Transcriptional changes linked to OA focus on immune control and microenvironmental

Interaction Pathways To explore the biological themes that elucidate the consistent transcriptional alterations seen in osteoarthritic cartilage, we conducted an enrichment analysis of the differentially expressed genes (DEGs) utilizing Gene Ontology (GO) and the Kyoto Encyclopedia of Genes and Genomes (KEGG). Rather than being scattered across various unrelated cellular functions, these genes exhibited a pronounced tendency towards activities associated with immune responses and interactions within the microenvironment, such as chemotaxis, taxis, cell-cell adhesion, promotion of cell adhesion, and regulation of leukocyte growth (**Figure 1C**). At the level of cellular components, the enriched terms were primarily linked to the outer regions of the plasma membrane, collagen-rich extracellular matrix, and the membranes of secretory granules, underscoring their significant role in communication between cells and between cells and the matrix. Additionally, the terms related to molecular functions encompassed immune receptor activity, integrin binding, extracellular matrix interaction, and protein-glycan binding, thereby emphasizing the concept of modified immune-matrix interactions in cartilage affected by OA.

The results of the KEGG pathway analysis reveal a notable enrichment in signaling pathways crucial for the regulation of the immune system and the microenvironment. This encompasses pathways such as the PI3K-Akt signaling pathway, interactions between cytokines and receptors, as well as cell adhesion molecules (CAMs) (**Figure 1D**). Overall, these enrichment findings suggest that immune regulation, the relay of inflammatory signals, and interactions with the extracellular matrix are fundamental biological processes that initiate transcriptional changes associated with OA. This underscores the importance of a network-based approach to pinpoint essential regulatory genes that govern these mechanisms.

### 2.3 Analysis of weighted gene co-expression networks reveals a gene module linked to OA

**Figure 2:**
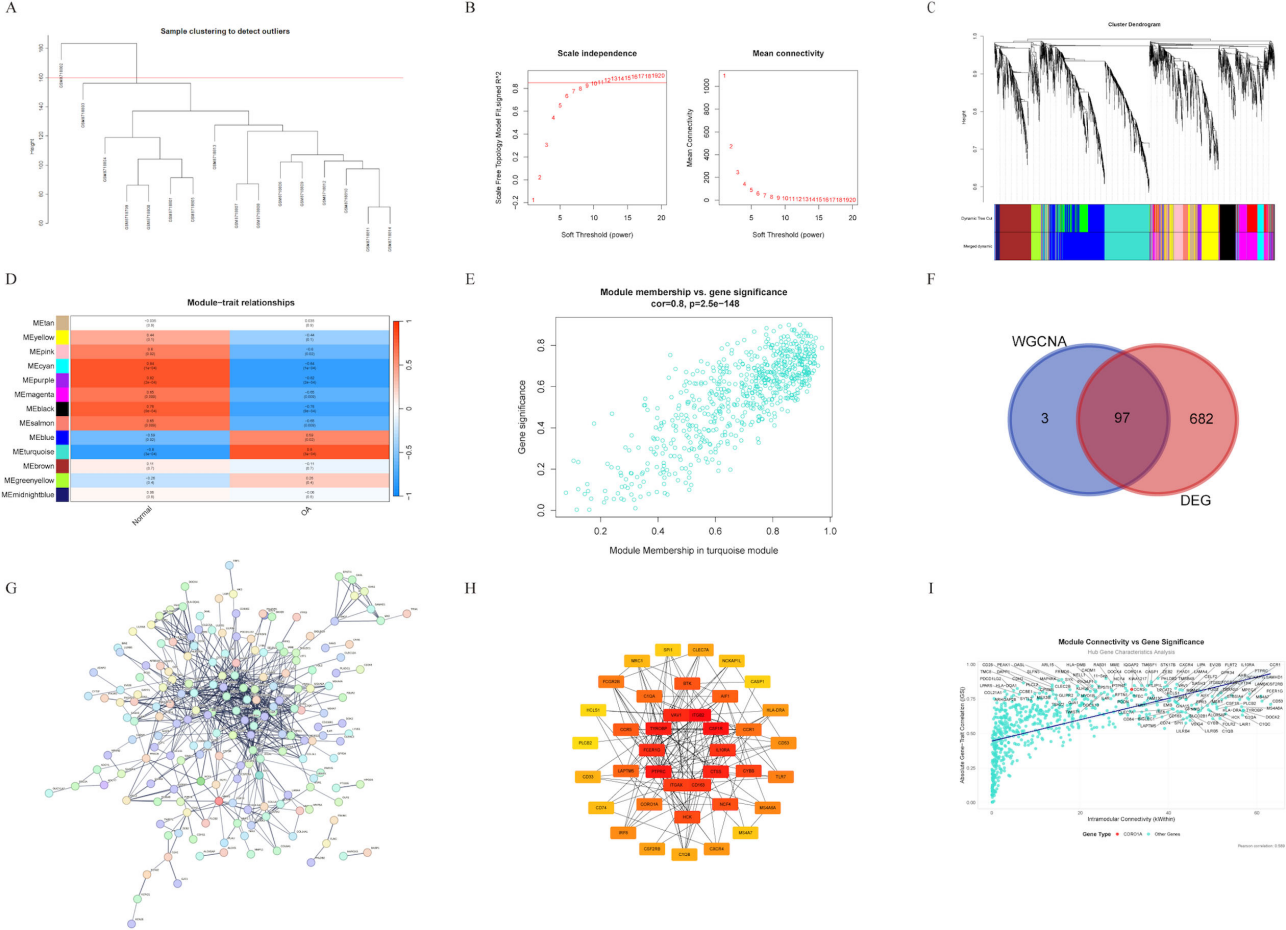
Identification and validation of key genes in osteoarthritis. **(A)** Clustering tree depicting eight Control samples alongside eight OA samples. **(B)** Evaluation of the scale-free fit index (left) and average connectivity (right) for a range of soft-thresholding powers. **(C)** Dendrogram displaying gene clusters arranged according to the dissimilarity of the topological overlap matrix, with color bands denoting recognized co-expression modules. **(D)** Heatmap illustrating correlations between module eigengenes and the OA phenotype. **(E)** Scatter plot comparing gene significance for OA to module membership within the turquoise module. **(F)** Venn diagram representing the intersection of 778 differentially expressed genes with the top 100 hub genes identified from the turquoise module. **(G)** Network of protein-protein interactions for the overlapping genes constructed using the STRING database. **(H)** Candidate hub genes identified by the CytoHubba plugin based on betweenness and stress centrality metrics. **(I)** Analysis of the relationship between intramodular connectivity (kWithin) and absolute gene significance (|GS|) across all modules, with CORO1A distinctly highlighted (Pearson r = 0.569).

In order to investigate the coordinated gene expression patterns relevant to osteoarthritis, we developed a weighted gene co-expression network utilizing the complete expression dataset from GSE286154. The initial evaluation involved clustering the samples to assess data quality and identify any potential outliers (**Figure 2A**). This analysis revealed that a topology could be established, achieving an approximate scale-free property with R^2^>0.85 at a soft-thresholding power of β=11, which facilitated the formation of a biologically meaningful network with sufficient gene connectivity (**Figure 2B**). We performed gene clustering based on topological overlap to create hierarchical co-expression modules, each represented by distinct colors (**Figure 2C**). To pinpoint the modules relevant to the disease, we analyzed the correlations between module eigengenes and the osteoarthritis phenotype. Among the modules examined, the turquoise module exhibited the strongest positive correlation with osteoarthritis (**Figures 2D, E**), suggesting that the genes within this module may be crucial in the molecular processes of OA. As a result, this module was chosen for further investigation of hub genes and their functional roles.

### 2.4 Identification of CORO1A as a potential gene linked to OA via network-centric hub gene evaluation

Recognizing that transcriptional alterations linked to osteoarthritis (OA) are associated with immune response pathways and interactions within the microenvironment, we aimed to pinpoint the crucial regulators that might orchestrate these processes at the network level. By integrating intramodular connectivity (kWithin) with gene significance (GS), we identified the top 100 hub genes and cross-referenced them with previously identified differentially expressed genes (DEGs), resulting in 97 common candidates (**Figure 2F**). This method of overlap ensured that our subsequent analyses concentrated on genes that were not only significant within the co-expression network but also exhibited transcriptional dysregulation during OA.

To clarify the functional relationships among the selected candidates, we developed a protein-protein interaction (PPI) network utilizing the STRING database. Analyzing the network’s topology showed a high degree of interconnectivity at its core, indicating the presence of essential regulatory nodes. By employing the CytoHubba plug-in, we ranked the genes based on two criteria: betweenness centrality and stress centrality, which assess the gene’s capacity to influence information flow within the network. The overlap of the top 40 genes identified through both metrics resulted in a collection of 39 reliable hub candidates (**Figure 2H**).

**Figure 3:**
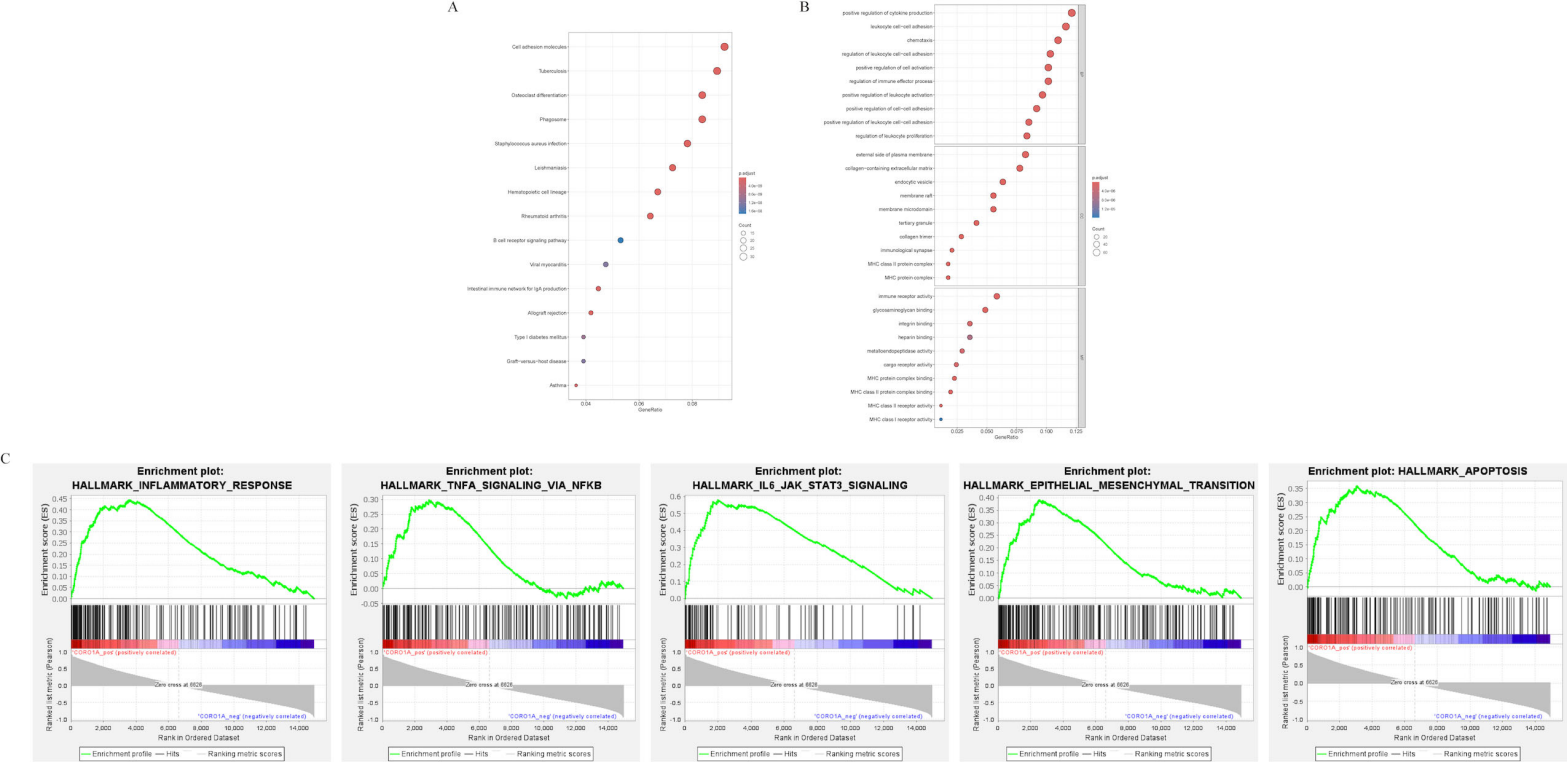
Analysis of functional enrichment for co-expressed genes located near CORO1A and a single-gene GSEA enrichment evaluation. **(A)** KEGG and **(B)** GO co-expressed genes around CORO1A enrichment analysis. **(C)** GSEA revealed that there was a remarkable increase in the inflammatory remodeling hallmark pathways e.g. IL6-JAK-STAT3 signaling, inflammatory response, epithelial-mesenchymal transition, TNF-α -NF-κB signaling and apoptosis in samples with an increase in CORO1A expression.

Among the potential hub candidates, CORO1A stood out due to its significant concentration of various analytical metrics, such as its position within a module, its connections to other nodes, and its association with diseases. Among the potential hub candidates, CORO1A stood out due to its significant concentration of various analytical metrics, such as its position within a module, its connections to other nodes, and its association with diseases. Notably, at the pathway level, these genes showed enrichment in immune and inflammatory pathways, encompassing phagosome, cell adhesion molecules (CAMs), systemic lupus erythematosus, rheumatoid arthritis, and graft-versus-host disease (**Figure 3A**). Furthermore, a functional enrichment analysis revealed that CORO1A and its co-expressed neighbors were significantly associated with immune-related biological processes, including leukocyte proliferation, modulation of immune effector processes, leukocyte-mediated immunity, monocyte proliferation, and phagocytosis (**Figure 3B**).

In summary, the findings from the network analysis highlight CORO1A as a crucial hub linking immune activation, inflammatory signaling, and cell interaction pathways that play a significant role in osteoarthritis progression. Due to its prominent centrality within the network, consistent differential expression, and potential role in immune-related joint disorders, CORO1A has been identified as the primary candidate gene for further experimental investigation.

### 2.5 CORO1A high expression correlates with pro-inflammatory and pro-fibrotic signaling programs

In order to explore the biological processes associated with elevated CORO1A expression, we performed a comparative analysis of global pathway activities in osteoarthritis (OA) samples exhibiting high versus low levels of CORO1A through Gene Set Enrichment Analysis (GSEA). Rather than identifying individual pathway alterations, our findings revealed that increased CORO1A expression was consistently associated with a synchronized upregulation of inflammatory responses, stress-related mechanisms, and tissue remodeling activities.

Among the gene sets that showed the highest enrichment in samples exhibiting elevated CORO1A levels, notable pathways included IL6-JAK-STAT3 signaling (NES = 2.83, FDR < 0.001), Inflammatory Response (NES = 2.49, FDR < 0.001), and TNF-alpha signaling through NF-κB (NES = 1.68, FDR < 0.001) (**Figure 3C**). Additionally, pathways associated with structural changes and fibrotic activation were also prominently enriched, such as Epithelial-Mesenchymal Transition (NES = 2.24, FDR < 0.001) and Apoptosis (NES = 1.97, FDR = 0.001). The simultaneous activation of inflammatory and mesenchymal-like pathways indicates that elevated CORO1A expression is associated not only with immune responses but also with catabolic and pro-fibrotic cellular conditions linked to cartilage damage and joint remodeling.

In summary, the findings across the entire system suggest that the expression of CORO1A is useful for categorizing OA samples within a distinct spectrum characterized by pro-inflammatory and pro-remodeling traits, providing a mechanistic understanding of its link to immune infiltration and the severe disease characteristics noted in subsequent evaluations.

### 2.6 Single-cell transcriptomics reveals preferential enrichment of CORO1A in inflammatory and proliferative chondrocyte subpopulations

In order to investigate whether the increased expression of CORO1A noted in large-scale transcriptomic studies of osteoarthritic cartilage indicates specific regulatory mechanisms at the single-cell level, we analyzed single-cell RNA sequencing (scRNA-seq) data from both normal and osteoarthritic cartilage samples (GSE169454). Following rigorous quality control measures, we identified 52,374 high-quality cells for subsequent analysis.

**Figure 4:**
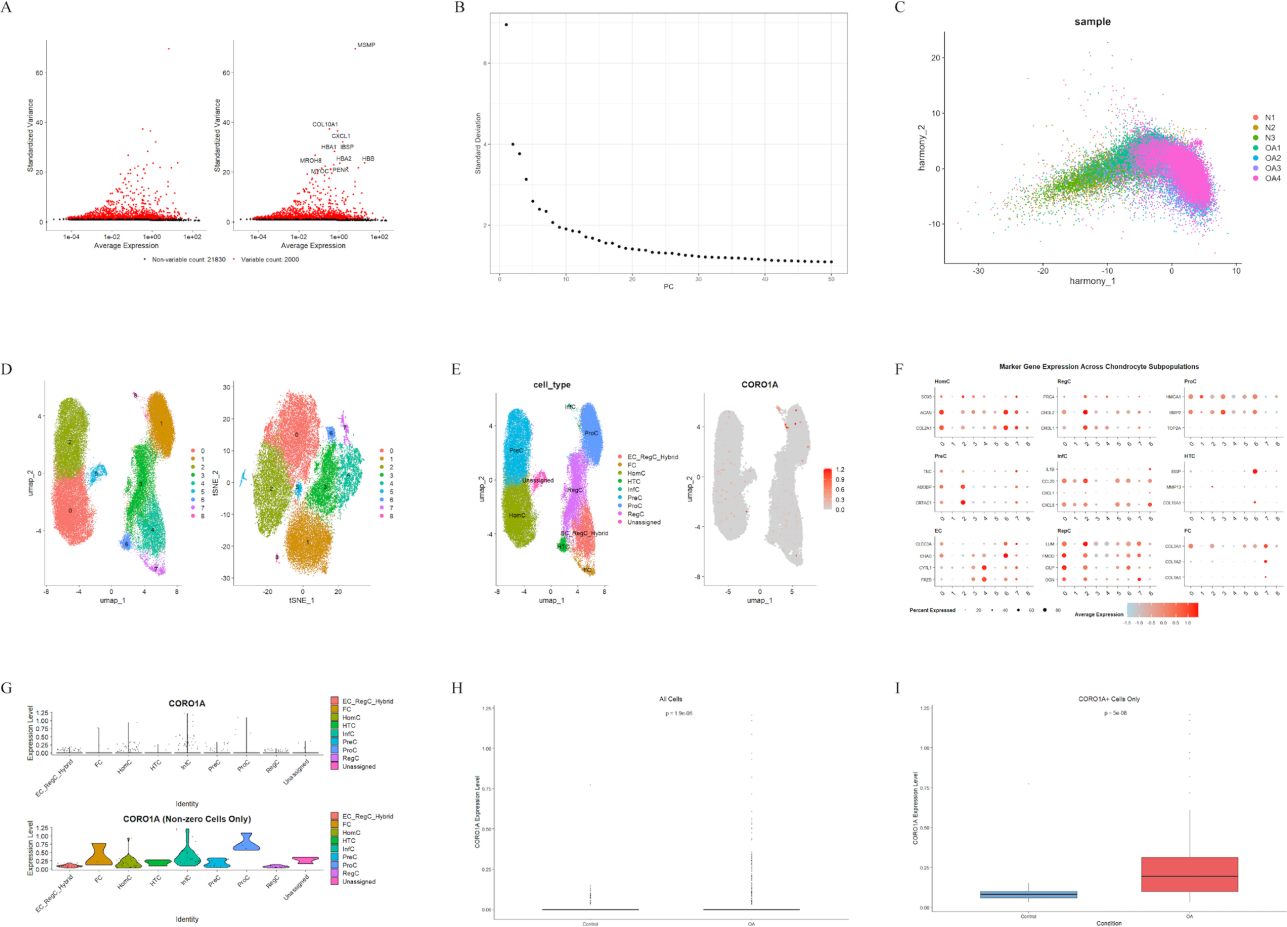
Single-cell transcriptomic landscape of normal and osteoarthritic (OA) cartilage. **(A)** 52,374 cells and 23,830 genes remained to analyze, on the basis of quality control: the 2,000 most highly variable genes (HVGs) were chosen in the top 2,000. **(B)** Elbow plot: visual plot used to calculate the dimensionality (number of principal components) to be used in downstream analysis. **(C)** Visualization of batch effect correction across samples using harmony integration. **(D)** Non-linear dimensionality reduction visualizations of the integrated dataset via UMAP (left) and t-SNE (right), revealing 9 transcriptionally distinct cell clusters. **(E)** The cell type annotation on UMAP projections (left) and expression of CORO1A (right) pattern. **(F)** Dot plot of the canonical marker genes of the annotated cell types. **(G)** Expression of CORO1A in the subpopulations of chondrocytes. Violin plots demonstrate the distribution in all the cells (top) and in the CORO1A+ cells (bottom) only. clusters InfC (inflammatory), HTC (hypertrophic-like), ProC (proliferating), RegC (regulatory), HomC (homeostatic), PreC (precursor), FC (fibrocartilage). **(H,I)** Comparison of CORO1A expression level in the Control group and OA group in all cells **(H)** and in CORO1A+ cells alone **(I)**. Box plots display interquartile range and median; p-values are displayed (Wilcoxon rank-sum test).

Through the application of unsupervised clustering techniques, we have successfully identified nine major groups of transcriptionally unique cell populations within the dataset, with chondrocytes emerging as the predominant cell type (**Figures 4B–D**). In the chondrocyte compartment, we recognized eight distinct subpopulations based on the expression of canonical marker genes and automated annotation methods. These subpopulations include inflammatory chondrocytes (InfC), proliferating chondrocytes (ProC), hypertrophic-like chondrocytes (HTC), homeostatic chondrocytes (HomC), precursor chondrocytes (PreC), fibrocartilage chondrocytes (FC), along with several other minor groups (**Figures 4E, F**).

Crucially, the expression of CORO1A demonstrated a notable selectivity for specific cell types, with a significant lack of enrichment in the chondrocyte population (**Figure 4E**). Upon examining various chondrocyte subtypes, it became evident that the expression levels of CORO1A were markedly elevated in these specific subtypes, which are known to contribute to inflammation associated with osteoarthritis (OA) and impaired cartilage remodeling. Additionally, our findings revealed that CORO1A was expressed at much higher levels in OA samples compared to control samples, both in the overall chondrocyte population and within the subset of cells that express CORO1A (**Figures 4H, I**).

Investigation on individual cells has revealed that the expression of CORO1A in osteoarthritis varies among chondrocytes, being particularly elevated in certain inflammatory and proliferative subgroups. This indicates that CORO1A plays a role in activating chondrocytes that contribute to the progression of OA.

### 2.7 CORO1A is elevated in degenerated cartilage found in human OA and experimental models

**Figure 5:**
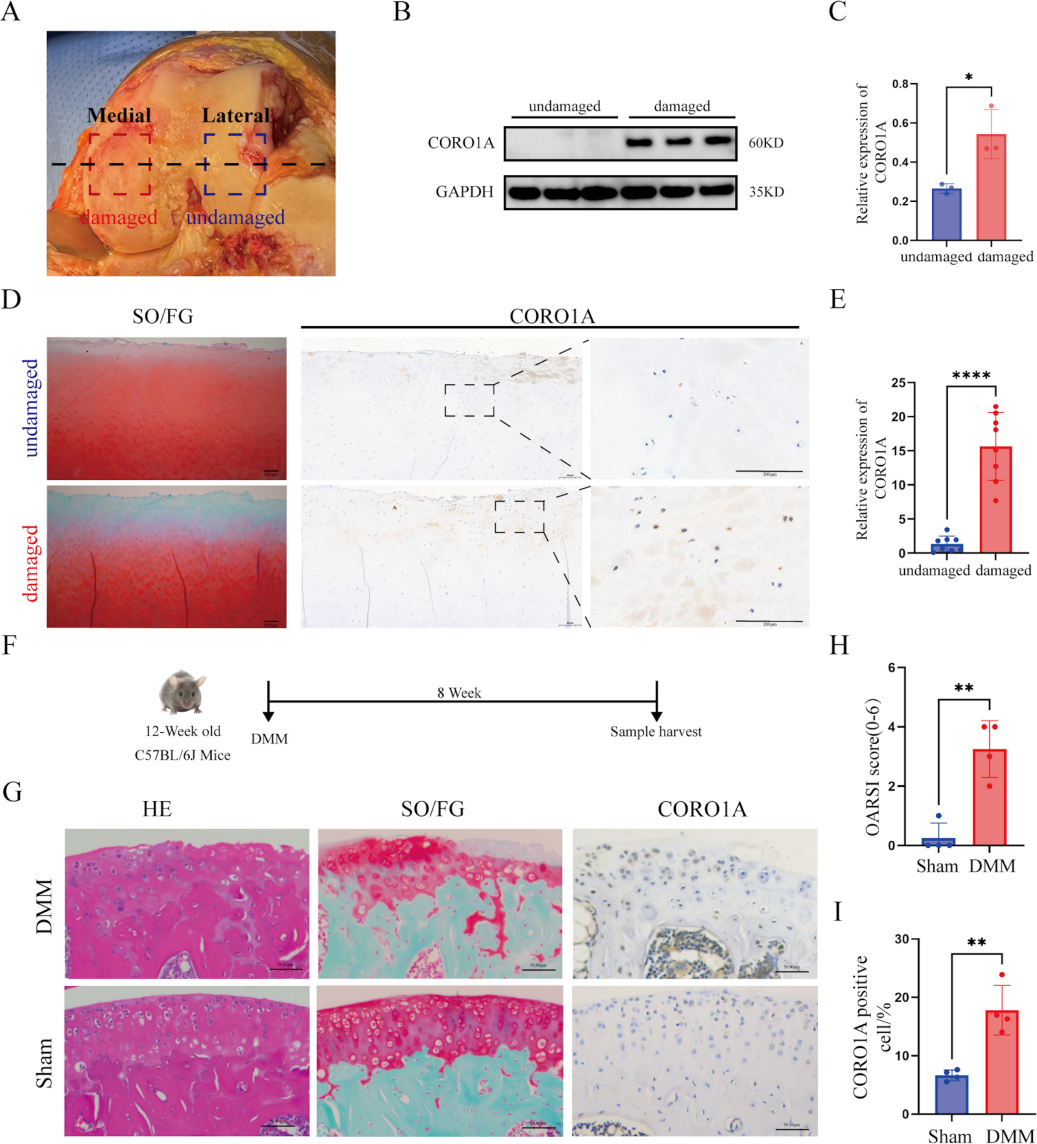
CORO1A is upregulated in osteoarthritic cartilage. **(A)** Cartilage tissues of the tibial plateau in patient-based schematic diagram. **(B)** CORO1A western blotting in comparatively intact and damaged cartilage. **(C)** Gray value analysis of Western blotting as in **(B)**, n=3 in each group. **(D)** Representative images of Safranin O/Fast Green staining of human OA articular cartilage and CORO1A immunohistochemical (IHC) staining of serial sections of human OA articular cartilage. **(E)** Quantification of CORO1A-positive chondrocytes as a percentage of total chondrocytes in intact cartilage and damaged cartilage of OA disease cases. Scale bars: 100 μm. n=8 per group. **(F)** Schematic illustration of experimental design of DMM mice. **(G)** HE staining, Safranin O/Fast Green staining, and immunohistochemical (IHC) staining of CORO1A of DMM mice articular cartilage representative images were used. **(H)** Statistical analysis of OARSI scores. **(I)** CORO1A-positive chondrocytes were analyzed quantitatively, according to the SO/FG and IHC staining. n=4 in every group. *p < 0.05, ** p < 0.01, ***p < 0.001, **** p < 0.0001.

To confirm the cellular findings from single-cell analysis within the tissue context, we assessed the natural expression of CORO1A in the cartilage of individuals with osteoarthritis and in a mouse model of the disease. Cartilage samples were obtained from patients undergoing total knee replacement surgery, with areas of intact and damaged cartilage identified based on anatomical location and visual characteristics (**Figure 5A**). Western blot analysis of these samples revealed a marked increase in CORO1A protein levels in damaged cartilage (**Figures 5B, C**). Consistently, immunohistochemical staining demonstrated a notable increase in the number of CORO1A-positive chondrocytes in the damaged regions compared to less affected areas (**Figure 5D**).

To validate these findings in a controlled setting, we employed the destabilization of the medial meniscus (DMM) mouse model. As expected, the DMM procedure led to significant cartilage deterioration and a reduction in proteoglycans, as evidenced by elevated OARSI scores (**Figure 5G**). Importantly, both conditions of cartilage affected by DMM exhibited a considerable increase in the proportion of CORO1A-positive chondrocytes compared to control samples, indicating that the presence of CORO1A-positive chondrocytes in degenerated cartilage is a notable aspect of osteoarthritis pathology (**Figures 5H, I**). In summary, the results indicate that the upregulation of CORO1A is a consistent observation in both human osteoarthritis and the mouse model.

### 2.8 CORO1A expression relates to a pro-inflammatory immune microenvironment in OA

**Figure 6:**
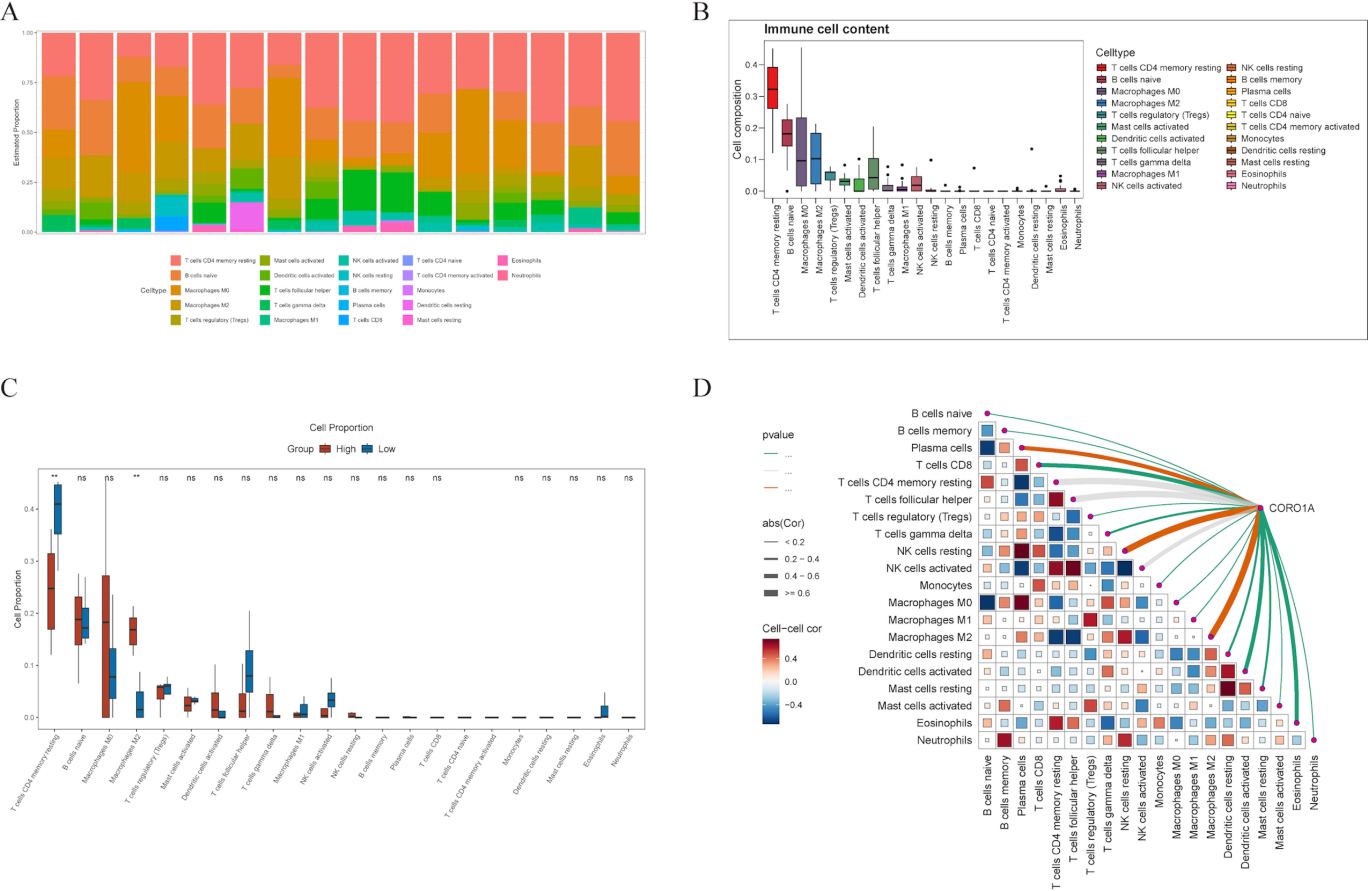
Correlation between immune cell infiltration. **(A)** Stacked bar plot showing the comparative proportion of 22 immune cell types in each sample. **(B)** Boxplot showing the overall composition of immune cell types within the cohort, with immune cell subtypes enumerated on the horizontal axis. **(C)** Box plot of immune cell proportions between high and low CORO1A expression samples. **(D)** Correlation network analysis of the association of immune cell subtypes to CORO1A gene expression. Those correlation coefficients are represented in the form of the adjacency matrix (color-coded blue positive correlation, red negative correlation, intensity proportional to magnitude). Significant statistical associations are represented as a network (orange edges: positive correlation; green edges: negative correlation; edge thickness proportional to correlation strength). The legend summarizes statistical significance levels and effect size, with Cor representing the absolute correlation coefficient. ** p < 0.01, ns = not significant (p ≥ 0.05).

Given the notable connections between CORO1A levels and inflammatory states in chondrocytes, we proceeded to investigate if CORO1A levels are linked to overall alterations in the immune microenvironment within osteoarthritic joints. We analyzed the proportions of 22 immune cell subtypes in osteoarthritis samples through CIBERSORT deconvolution applied to bulk transcriptomic data (see **Figures 6A, B**). The immune structure of OA cartilage exhibited considerable diversity, predominantly featuring macrophages, T cells, and B cells.

When analyzing lineage cells, significant differences in immune cell composition emerged based on CORO1A expression levels. Notably, samples with elevated CORO1A expression exhibited a marked rise in the infiltration of M2 macrophages, plasma cells, and activated NK cells, while showing a decrease in naive B cells and inactive mast cells (**Figures 6C, D**). These immune cell subsets are crucial for chronic inflammation, antibody-driven immune responses, and tissue remodeling in osteoarthritis. This finding reinforces the connection between CORO1A and an enhanced immune environment that favors osteoarthritis, highlighting its role as a molecular link between chondrocyte dysfunction and immune-related pathology.

### 2.9 CORO1A enhances catabolic behavior and chondrocyte mobility in inflammatory settings

**Figure 7:**
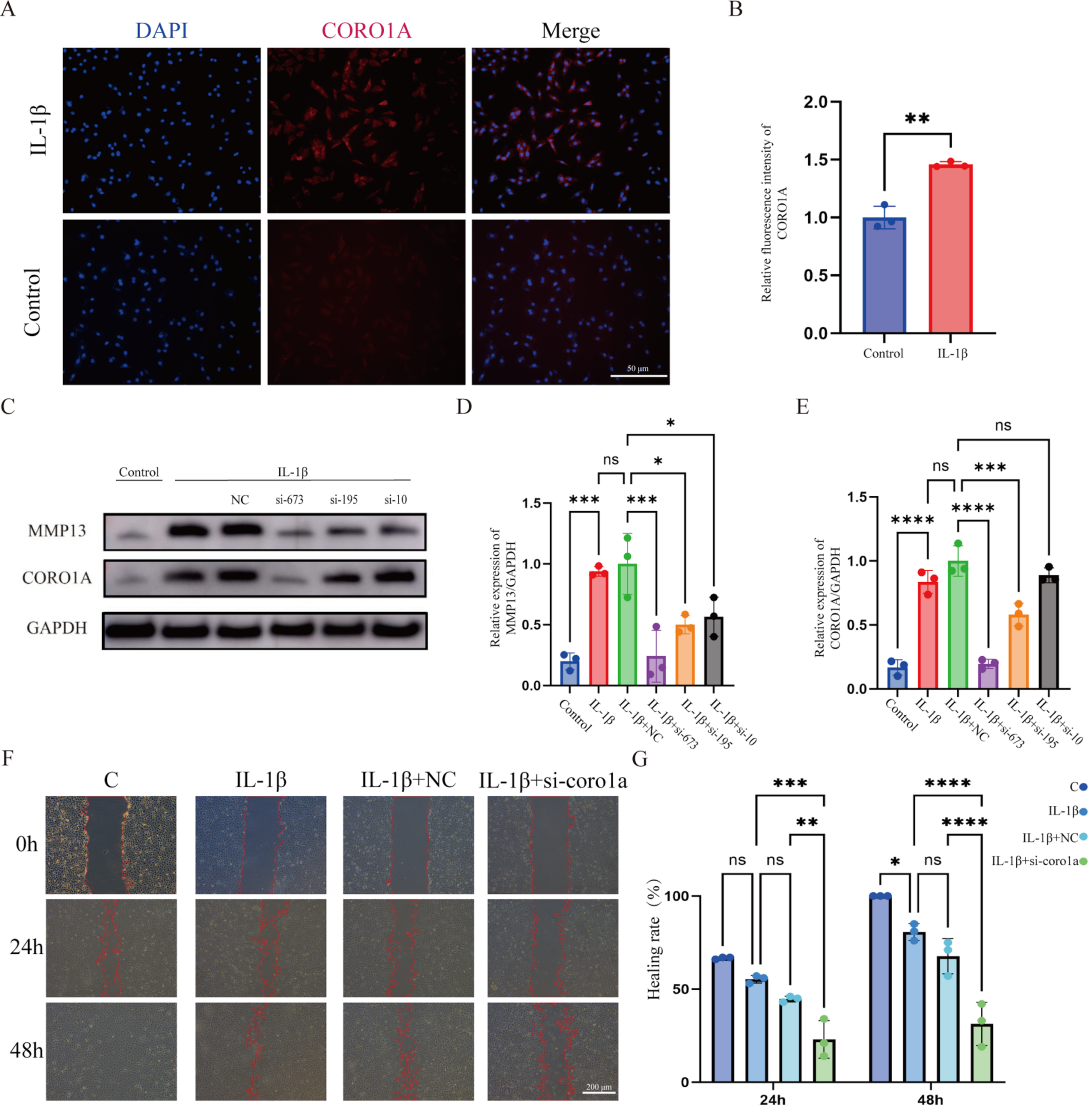
CORO1A promotes catabolic response and migration in primary chondrocytes. **(A)** Control and IL-1β-treated chondrocyte representative immunofluorescence images of CORO1A (red) and nuclei (DAPI, blue). **(B)** CORO1A mean fluorescence intensity quantitative analysis. n=3/group. Scale bars: 50μm. **(C)** To examine CORO1A and MMP13 in chondrocytes stimulated with IL-1β and transfected with negative control siRNA (NC) or CORO1A-target siRNAs (si-673, si-195, si-10), a CORO1A Western blotting experiment was carried out using the GAPDH as the control. **(D)** MMP13 protein levels of **(C)**. **(E)** CORO1A protein levels in **(C)**. **(F)** Representation of the wound healing test at 0, 24, and 48 h, scale bar: 200μm. **(G)** Quantification of wound closure rates. n=3/group. *p < 0.05, ** p < 0.01, ***p < 0.001, **** p < 0.0001, ns = not significant.

To investigate the function of CORO1A in chondrocyte behavior, we employed an inflammatory model using IL-1β in primary mouse chondrocyte cultures. Immunofluorescence analysis revealed a notable increase in CORO1A protein levels following IL-1β treatment (**Figures 7A, B**). Notably, the reduction of CORO1A resulted in a significant decline in MMP13 expression induced by IL-1β (**Figure 7D**). To confirm CORO1A’s active involvement in these processes, we conducted a knockdown using siRNA. Among the three siRNAs evaluated, si-673 proved to be the most effective in diminishing CORO1A expression and was chosen for subsequent experiments (**Figure 7E**).

The involvement of CORO1A in the catabolic processes of chondrocytes during inflammation was investigated. Given that CORO1A regulates the cytoskeletal structure, we assessed its impact on chondrocyte movement. Our wound-healing assays revealed a notable reduction in the migration ability of chondrocytes with CORO1A knockdown at both the 24 and 48-hour marks post-scratch injury (**Figures 7F, G**).

In summary, our findings suggest that CORO1A acts as a mediator of catabolism and aids in the movement of chondrocytes during inflammation, thereby reinforcing the theory that CORO1A promotes harmful cellular processes associated with the development of osteoarthritis.

## 3 Discussion

Osteoarthritis (OA) is acknowledged as a widespread joint disorder characterized by cartilage degradation, synovial inflammation, and subchondral bone alterations. To comprehend the reasons behind the inability of cartilage to sustain homeostasis as OA progresses, it is important to identify factors linking inflammatory signaling to changes in chondrocyte behavior (22, 23).

Our bioinformatics analysis identified immune-regulatory, cytokine-receptor, PI3K-Akt, and cell adhesion-related pathways as major features of osteoarthritic cartilage. These enrichment results suggest that OA-associated transcriptional changes are not driven solely by classical inflammatory cytokines such as IL-1β and TNF-α, but are also linked to cell survival, adhesion, extracellular matrix interactions, and potentially motility-related processes. Consistently, clinical trials of biologic anti-inflammatory agents in hand OA, including TNF inhibitors, have shown limited symptomatic efficacy, suggesting that OA-related inflammatory-catabolic responses cannot be fully explained by canonical cytokine-driven inflammation alone (24). In this context, CORO1A-related changes may represent part of a broader network linking inflammatory signaling, adhesion/cytoskeletal remodeling, and chondrocyte state changes, rather than merely a common inflammatory signature.

Our focus on CORO1A stems from the pivotal role that coronin proteins play in linking actin dynamics and signal transduction (14, 25). Although CORO1A has been extensively examined in immune contexts, particularly regarding its capacity to influence receptor signaling and cytoskeletal organization (26), its significance in the context of osteoarthritis (OA) cartilage remains inadequately defined. Our findings suggest that CORO1A may also be relevant to cartilage pathology beyond its established role in immune-related cytoskeletal regulation.

In both human osteoarthritic cartilage and the DMM model, a consistent increase in CORO1A levels was noted, indicating an association with a degenerative-inflammatory cartilage state. Notably, in certain DMM cases, a rise in CORO1A staining was observed alongside increased cartilage damage (see **Supplementary Figure 2**). While these instances were insufficient for subgroup analysis, these observations are consistent with an association between CORO1A expression and a more inflammatory or degenerative cartilage state. Supporting this notion, CORO1A has been detected in notably higher amounts in more systemically inflamed arthritic conditions, such as the synovial fluid of RA (20), implying that CORO1A might reflect an inflammatory response rather than being specific to a particular disease (12). This differentiation is crucial for its translational significance: CORO1A could serve as a more valuable indicator of the interactions between inflammatory and catabolic processes and cellular states, rather than merely as a diagnostic marker for a single condition.

Our scRNA-seq analysis further refined this observation by showing that CORO1A was not uniformly elevated across all chondrocytes. Instead, its up-regulation is specifically observed in two disease-relevant states: inflammatory chondrocytes (InfC) and proliferative chondrocytes (ProC). This pattern suggests that CORO1A is associated with specific disease-related chondrocyte states rather than generalized cartilage degeneration (27).

The presence of CORO1A in both InfC and ProC raises the possibility that persistent inflammatory signals may engage a cytoskeleton-linked signaling program associated with both catabolic gene expression and cell motility. As a member of the coronin family and an actin-binding signaling molecule, CORO1A may act at the interface of membrane dynamics and signal transduction, providing a plausible link to cytokine responsiveness and cell motility (25, 28).

*In vitro*, siRNA-mediated CORO1A knockdown attenuated IL-1β-induced MMP13 expression and reduced scratch closure, supporting a possible association between CORO1A and inflammatory-catabolic as well as motility-related responses in chondrocytes. Mechanistically, this may involve small GTPase signaling, as CORO1A has been reported to scaffold Rac-family signaling complexes and Rac signaling is known to regulate cytoskeletal rearrangement and cell migration (14). Further studies are needed to determine whether Rac activation and downstream actin dynamics mediate CORO1A-associated catabolic and migration-related responses under inflammatory stimulation.

Immune infiltration analysis showed that samples with high CORO1A expression exhibited increased infiltration of M2 macrophages. Although M2 macrophages are generally considered anti-inflammatory and repair-associated, their functions in chronically inflamed tissues may be highly context-dependent; persistent M2-like polarization does not necessarily indicate effective inflammation resolution or tissue repair, but may instead reflect dysregulated repair, immune remodeling, or unresolved inflammation. This association may involve both macrophage-intrinsic and microenvironment-dependent mechanisms. One testable explanation is that CORO1A may be linked to TGF-β/SMAD3-related signaling, as TGF-β is an important inducer of M2-like macrophage polarization and Coronin 1A has been reported to regulate TGF-β receptor/SMAD3 signaling in Th17 CD4+ T cells (26, 29). In addition, studies in other disease contexts have linked CORO1A to PI3K/AKT/mTOR-related signaling and myeloid cell regulation (30, 31), providing indirect support for a possible connection between CORO1A-associated signaling programs and macrophage polarization. Alternatively, CORO1A-high samples may reflect broader cytokine- and chemokine-associated inflammatory programs, including pathways related to NF-κB or IL-6/STAT3 signaling (32, 33), which could indirectly influence macrophage recruitment or polarization by reshaping the local inflammatory microenvironment. However, the current data cannot distinguish whether increased M2 macrophage infiltration reflects macrophage-intrinsic CORO1A activity, paracrine signals derived from CORO1A-high chondrocyte states, or both. Therefore, the association between CORO1A expression and M2 macrophage infiltration should be interpreted as hypothesis-generating rather than causal.

Several limitations should be acknowledged. First, the scRNA-seq dataset is based on a limited number of samples, restricting generalizability. Second, the *in vitro* model reflects acute cytokine stimulation and does not capture chronic disease dynamics. Third, the immune infiltration analysis is correlative and does not establish causality. Fourth, the absence of a matched isotype control limits definitive assessment of the specificity of immunofluorescence staining. Finally, the use of macroscopically unaffected cartilage as a control may not fully represent true healthy tissue, as such regions may already exhibit molecular alterations.

In addition, while this study focuses on ECM catabolic processes, impaired matrix synthesis is also a critical component of OA progression. Future studies are needed to determine whether CORO1A affects anabolic pathways, including COL2 and Aggrecan expression.

In conclusion, our study identifies CORO1A as a context-associated regulator linked to inflammatory signaling and chondrocyte state remodeling in OA. The selective enrichment of CORO1A in inflammatory and proliferative chondrocytes suggests that OA progression is associated with the activation of specific CORO1A-high pathogenic programs rather than uniform activation across all chondrocytes. Through multi-omics analysis and functional perturbation, we propose that CORO1A represents a testable component of a cartilage-immune network associated with inflammatory-catabolic responses, providing a basis for further mechanistic studies.

## 4 Methods

### 4.1 Data acquisition

Transcriptomic datasets related to osteoarthritis (OA) and cartilage were sourced from the Gene Expression Omnibus (GEO) database (https://www.ncbi.nlm.nih.gov/geo/). For this analysis of transcriptional changes in OA-affected tissues, two specific datasets were selected. One of these is the bulk cartilage gene expression dataset, GSE286154, which involves a paired-sample approach utilizing 16 cartilage specimens from eight OA patients. Each patient provided cartilage samples from both less affected areas, which were relatively smooth, and more severely damaged regions that bear greater weight. The classification of these samples was guided by the Mankin scoring system, a method that was subsequently employed for expression and network analysis differentiation.

After collecting and reviewing the data, we selected the single-cell RNA sequencing dataset GSE169454 (34) for our analysis of cellular diversity. Ultimately, we investigated the expression patterns and distribution of the CORO1A gene across different chondrocyte subpopulations in individual samples from both osteoarthritis patients and non-osteoarthritis controls, focusing on articular cartilage specimens.

### 4.2 Analysis of differential expression and functional enrichment

The analysis of differential gene expression was conducted utilizing the limma R package (35) which compared samples of damaged cartilage to those of intact cartilage from the same osteoarthritis patients in the GSE2861454 dataset. To address the paired nature of the study, patient identities were included as a blocking factor in the linear model. Genes were deemed significantly differentially expressed if they had an adjusted P-value (using the Benjamini-Hochberg method) below 0.05 and an absolute log2 fold change exceeding 1.896. Visualization of the differentially expressed genes (DEGs) was carried out using the pheatmap and ggplot2 packages. Following this, enrichment analyses for Gene Ontology (GO) and Kyoto Encyclopedia of Genes and Genomes (KEGG) pathways were performed with the clusterProfiler package, considering a significance threshold of less than 0.05.

### 4.3 Gene set enrichment analysis

We categorized the gene expression profiles based on CORO1A levels into two groups: CORO1A-low and CORO1A-high, using the median as a threshold. Phenotypic data were organized in CLS format, while mRNA expression data were compiled in GCT format. Subsequently, we conducted Gene Set Enrichment Analysis (GSEA) (36) on the differentially expressed genes and associated signaling pathways, utilizing Hallmark gene sets from the MSigDB database. The gene sets of particular interest were those that exhibited a normalized enrichment score (NES) with an absolute value exceeding 1.5 and a false discovery rate (FDR) below 0.05.

### 4.4 Identification of OA-related modules with WGCNA

Gene expression data underwent normalization and correction through the NormalizeBetweenArrays algorithm. Subsequently, a weighted gene co-expression network was constructed utilizing the WGCNA package in R. The soft-thresholding power was set to 11, achieving a scale-free topology with the highest R2 value. Gene hierarchical clustering was performed using a topological overlap matrix (TOM) to assess pairwise dissimilarity, employing average linkage and dynamic tree cutting methods. Module definition was based on a minimum of 75 genes, and modules with similar expression patterns (height cut-off = 0.25) were merged. Genes within the same module exhibited high topological overlap, indicating co-expression. Significant correlations with osteoarthritis (OA) traits were identified, and the modules with the strongest associations were selected for further investigation. To visualize the relationship between module membership (inter-module connectivity) and gene significance in OA, scatter plots were created for the key modules of interest (**Supplementary Table 3**), with the connection quantified using the Pearson correlation coefficient. Hub genes within a module were identified as those with high intramodular connectivity and significant relevance to OA. Finally, Gene Ontology (GO) and Kyoto Encyclopedia of Genes and Genomes (KEGG) pathway enrichment analyses were performed on the most relevant OA module, the CORO1A gene, along with its direct neighbors, using the cluster Profiler R package.

### 4.5 Construction of protein-protein interaction network and identification of hub genes

A network of protein-protein interactions (PPI) was created to elucidate the functional relationships among the differentially expressed genes (DEGs) and identify key hub genes. The DEGs were submitted to the STRING database (version 11.5), where only interactions with a minimum required score of 0.7 were retained to ensure high-quality data. This interaction data was then imported into Cytoscape software (version 3.9.1) for network visualization. The CytoHubba plug-in was employed to calculate centrality metrics for each node within the network. Gene rankings were primarily determined using two topological algorithms: Betweenness and Stress centrality. The top 40 genes from each ranking were selected, resulting in an intersection of 39 high-confidence candidate hub genes for further analysis. It is important to mention that the subsequent focused investigation of CORO1A was based on a hypothesis, as it has been previously linked to the pathophysiology of arthritis (20).

### 4.6 Immune infiltration analysis

The estimation of immune cell fractions was conducted using CIBERSORT (37). along with the LM22 signature matrix and involved 1,000 permutations. Only those samples that demonstrated a CIBERSORT deconvolution P-value of less than 0.05 were included in subsequent correlation analyses. The relationship between CORO1A expression and immune cell fractions was analyzed using Spearman correlation. To ascertain the immune cell fractions through CIBERSORT, the analysis utilized 1,000 permutations with the LM22 signature matrix. Samples meeting the criteria of a CIBERSORT deconvolution P-value below 0.05 and the 1,000 permutations were retained for further correlation studies. The connections between CORO1A expression and immune cell proportions were assessed via Spearman correlation.

### 4.7 Single-cell transcriptomics identifies OA-associated chondrocyte subpopulations enriched for CORO1A

The GSE169454 dataset underwent processing and analysis of single-cell RNA sequencing (scRNA-seq) utilizing the Seurat R package (version 5.0+). Initially, a quality control (QC) procedure was applied to filter the dataset, ensuring that only genes expressed in a minimum of three cells were retained. Cells were selected based on having between 300 and 4,500 unique genes, while those with mitochondrial gene content exceeding 20% of the total were excluded. Gene expression counts were normalized using the logNormalization method. The top 2,000 highly variable genes (HVGs) were identified through the variance-stabilizing transformation (vst) technique in FindVariableFeatures (**Figure 4A**). Data scaling was performed with the ScaleData function, which also accounted for variations related to mitochondrial gene expression. Following this, principal component analysis (PCA) was conducted, and the optimal number of principal components for subsequent analysis was determined via an elbow plot. To mitigate potential batch effects on individual sample data, the R package Harmony was employed for further correction. Cell clustering was achieved by constructing a common nearest neighbor graph with the FindNeighbors function, followed by community detection using the FindClusters function at a resolution of 0.4. The resulting clusters were visualized in two dimensions using Uniform Manifold Approximation and Projection (UMAP). Finally, cell type annotations were performed by examining the expression of cluster-specific markers (identified with the FindAllMarkers function) alongside well-documented markers from existing literature.

### 4.8 Human samples

Cartilage tissues from individuals with osteoarthritis (OA) who were receiving total knee replacement surgery were obtained for this study. The research was sanctioned by the Ethical Review Board of the Second Affiliated Hospital of Guangxi Medical University (Approval No. 2024-KYL (017)). Clinical details of the donors are summarized in **Supplementary Table 2**. To facilitate comparisons between affected and unaffected cartilage while minimizing variations among patients, samples were gathered from both the lateral and medial tibial plateau. Following collection, the samples were either preserved in liquid nitrogen or fixed in 4% paraformaldehyde for subsequent analysis as required. Full-thickness cartilage underwent histological examination and immunohistochemistry (IHC). Additionally, the cartilage was minced and lysed using RIPA buffer containing protease inhibitors. Western blotting was employed to analyze the expression patterns of CORO1As in both damaged and intact regions.

### 4.9 Animal model

The mice utilized in this study were kept in the SPF-grade laminar flow facilities at the Experimental Animal Center of Guangxi Medical University. The temperature was consistently regulated at 25 ± 2 °C, with humidity levels ranging from 55% to 75%. They had unrestricted access to standard pellet food and water, and their bedding was replaced daily while their cages were regularly disinfected to maintain hygiene. To induce osteoarthritis (OA), we performed destabilization of the medial meniscus (DMM) surgery on 12-week-old mice, which involved making an incision in the capsule of the right knee and severing the medial meniscus-tibial ligament. Mice in the sham group underwent the same surgical procedures but did not have their ligaments cut. After an eight-week period, the mice were euthanized, and their knee joints were extracted for histological and immunohistochemical analysis, focusing on cartilage degeneration. This research received approval from the Ethics Committee of the Animal Care Committee at Guangxi Medical University (Approval No. 202411045).

### 4.10 Processing of tissue samples, histological examination, and immunohistochemical staining

Biochemical and histological evaluations of articular cartilage were performed using collected samples. Specimens from the knee joints were stored in 4% paraformaldehyde for 24 hours, followed by a decalcification process in 10% EDTA (pH 7.4) for 21 days (or 7 days for mouse knee samples, with the solution changed every 3 days). Subsequently, the samples underwent dehydration and were embedded in paraffin. Cross-sections were prepared at a thickness of 4 μm, and the condition of the articular cartilage was assessed using Safranin O and Hematoxylin and Eosin (H&E) staining. Images were captured using a Cytation5 microscope, and cartilage degradation was quantified using the Osteoarthritis Research Society International (OARSI) scoring system along with ImageJ software. Antigen retrieval was performed with citrate buffer, and endogenous peroxidases were inhibited using 3% H2O2 in methanol. Sections were blocked with goat serum and incubated overnight at 4 °C with a primary antibody against CORO1A (1:300, Cell Signaling Technology). Following this, a horseradish peroxidase (HRP)-conjugated goat anti-rabbit IgG polymer was applied, and the samples were stained with diaminobenzidine (DAB) and hematoxylin. Finally, the sections were dried, fixed, and scanned.

### 4.11 Extraction of primary cells lines, cell culture, biological reagents and transfection

The primary chondrocytes were extracted from the knee joint cartilage of C57BL/6 wild type mouse pups aged 0 to 3 days postnatally. This extraction involved digesting the cartilage tissue with a solution of type II collagenase.

After digestion, the isolated primary chondrocytes were re-suspended in DMEM/F12 medium supplemented with 10% fetal bovine serum, 50 U/mL penicillin, and 0.05 mg/mL streptomycin. To promote optimal growth, the cells were cultured in sterile monolayers at 37 °C in a humidified environment with 5% CO^2^. To ensure the reliability of the experimental outcomes, the chondrocytes were not subcultured beyond the third generation, preserving their viability for future studies. To establish an *in vitro* model of cartilage inflammation, the cultured chondrocytes were treated with 10 ng/mL recombinant mouse IL-1β for 24 hours, simulating an inflammatory setting to observe the chondrocyte response. Additionally, to explore the function of the Corola gene, the cells were transfected with double-stranded siRNA targeting Corola. All siRNA sequences (refer to **Supplementary Table 1**) were obtained from Sangon Biotech (Sangon, Shanghai, China). This experiment aimed to assess the effects of reduced Corola expression on chondrocyte functionality and inflammatory responses under the specified conditions.

### 4.12 Immunofluorescence staining

Chondrocytes were cultured on glass coverslips and subsequently fixed using a 4% paraformaldehyde solution (prepared in 0.1 M PBS, pH 7.4) for 15 minutes at ambient temperature. Following this, the cells were rinsed with PBS and permeabilized with 0.5% Triton X-100 for 10 minutes. A blocking step was performed using a 3% BSA solution for one hour. The cells were then incubated overnight at 4 °C with a rabbit antibody against CORO1A (Cell Signaling Technology) at a dilution of 1:100. After additional washing, the samples were treated with goat anti-rabbit IgG conjugated to Alexa Fluor 594 (1:500; ZSGB-BIO) under light-protected conditions at room temperature. Finally, the nuclei were stained with DAPI (1:5000; Sigma-Aldrich) for 10 minutes. The coverslips were mounted using an anti-fade medium and examined with an OLYMPUS IX73 microscope equipped with a 60x oil immersion objective and appropriate filter sets for detecting CORO1A and DAPI-stained nucleic acids.

### 4.13 Cell scratch assay

The scratch assay procedure begins with the growth of primary cells in six-well plates. After the cells have attached and multiplied, various treatments, such as the introduction of interleukin-1 and siRNA transfection, are applied according to the designated experimental groups. Finally, a wound gap is created by using a 200μL pipette tip to scratch the cell monolayer. The width of this wound is recorded at three time points: at the start of the experiment (0 hours), and then at 24 and 48 hours. Wound closure was monitored using phase-contrast microscopy without additional staining.

### 4.14 Western blot analysis

Cell lysis was performed using a lysis buffer created by combining protease inhibitors, phosphatase inhibitors, phenylmethylsulfonyl fluoride (PMSF), and RIPA buffer in a ratio of 1:1:1:1000. The lysate was centrifuged to clarify it, and the protein concentration in the pellet was measured using the bicinchoninic acid (BCA) assay. The protein samples were then mixed uniformly with SDS-PAGE loading buffer and subjected to SDS-PAGE electrophoresis under denaturing conditions, followed by transfer to polyvinylidene difluoride (PVDF) membranes. After blocking, the membranes were incubated overnight at 4 °C with primary antibodies: anti-CORO1A (1:1,000; Cell Signaling Technology, USA), anti-MMP13 (1:5,000; Proteintech, USA), and GAPDH (1:50,000; Proteintech, USA) as the internal control. Finally, the membranes were treated with a fluorescent secondary antibody (goat anti-rabbit IgG (H+L), DyLight ^®^ 800 4X PEG; Invitrogen, USA). The protein bands were thoroughly washed with TBST solution and imaged using the ImageQuant LAS-4000 system.

### 4.15 Statistical analysis

The quantification of immunohistochemical metrics, such as the percentage of stained areas and the proportion of positive cells, was conducted using QuPath software (version 0.6.0) (34). The analysis of the results was performed with R statistical software or GraphPad Prism (version 10.1.2). To determine statistically significant differences in mean expression values, either one-way ANOVA followed by Tukey’s *post-hoc* test or Student’s t-test was employed. A P value of less than 0.05 was deemed statistically significant.

## Data Availability

The original contributions presented in the study are included in the article/**Supplementary Material**. Further inquiries can be directed to the corresponding authors.
