# Supplementary material for: CORO1A links inflammatory chondrocyte subpopulations to immune microenvironment alterations in osteoarthritis: an integrative multi-omics and single-cell study
Source: Front Immunol. 2026 Jun 11;17:1820391. doi: 10.3389/fimmu.2026.1820391 (PMC13293819; doi:10.3389/fimmu.2026.1820391)
Supplement: Supplementary file 1 [file Table1.docx]

**SUPPIementary** **MateriaI**

**SUPPLEMENTARY** **DATA** **DESCRlPTloN**

This supplementary material includes:

**Table** **S1-8**

**Figure** **S1-3**

**1** **SUPPLEMENTARY** **TABLES** **AND** **FlGURES**

**Table** **S1.** siRNA sequences used in this study.

| **siRNA** **Name** | **sense** **(5,-3,)** | **antisense** **(5,-3,)** | **Efﬁciency** |
| --- | --- | --- | --- |
| si-coro1a-673 | CGCUGUGUUUGUGUCAGAANaT//aT/ | UUCUGACACAAACACAGCG/aT//aT/ | Most effective |
| si-coro1a-195 | AGACUGGACGAGUAGACAA/aT//aT/ | UUGUCUACUCGUCCAGUCU/aT//aT/ | secondary |
| si-coro1a-10 | GGUGGUUCGCUCCAGCAAANaT//aT/ | UUUGCUGGAGCGAACCACC/aT//aT/ | Tertiary |

Three distinct siRNA sequences targeting the mouse *coro1a* (NcBI Gene: 12721) mRNA and a negative control (Nc) siRNA with no signiﬁcant homology to any known human gene sequences were designed and synthesized by sangon Biotech (shanghai, china). All transfections were performed using the

transfection reagent supplied by sangon Biotech according to the manufacturer’s protocol, with a ﬁnal siRNA concentration of 50 nM. The knockdown

efﬁciency of each siRNA was validated by western blotting (Fig. 7c-E). si-cORO1A-673 was selected for all subsequent functional experiments due to its superior knockdown efﬁciency.

**Table** **S2.** clinical characteristics of Osteoarthritis patients

| **ID** | **Joint** | **Age** **(years)** | **Gender** | **Diagnosis** |
| --- | --- | --- | --- | --- |
| OA-001 | R | 68 | F | knee osteoarthritis |
| OA-002 | L | 72 | F | knee osteoarthritis |
| OA-003 | R | 70 | F | knee osteoarthritis |
| OA-004 | R | 68 | M | knee osteoarthritis |
| OA-005 | R | 72 | F | knee osteoarthritisa |
| OA-006 | R | 70 | F | knee osteoarthritis |
| OA-007 | R | 68 | F | knee osteoarthritis |
| OA-008 | R | 72 | F | knee osteoarthritis |
| **Summary** **Statistics**  Mean age: 70.0 ± 1.8 years  Gender: Female 7 (87.5%), Male 1 (12.5%)  Affected side: Right 7 (87.5%), Left 1 (12.5%) | | | | |

a Bilateral involvement, right side recorded as primary symptomatic side

**1.1** **Figures**


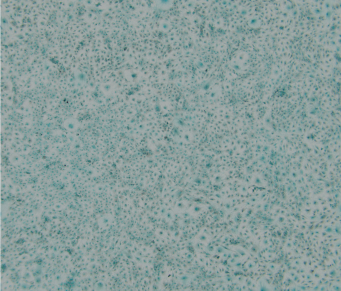
1000 μm

**Figure** **S1.** Chondrocyte identiﬁcation. primary chondrocytes from C57 neonatal mice show positive Alcian blue staining for proteoglycans, conﬁrming chondrogenic phenotype. scale bar: 1000 μm.

sham

HE


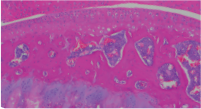


200.00μm

sO/FG


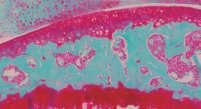


200.00μm

CORO1A


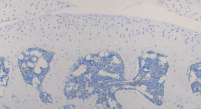
200.00μm

DMM


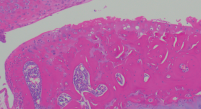


200.00μm


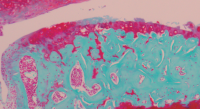


200.00μm


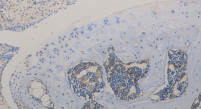


200.00μm

severe-OA


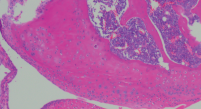


200.00μm


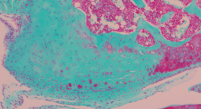


200.00μm


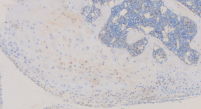


200.00μm

**Figure** **S2.** Comparative histology of sham, DMM, and severe-OA groups. Rows from top: HE staining (cartilage structure), sO/FG staining (proteoglycans), CORO1A IHC (protein expression). severe-OA shows spontaneous arthritis exacerbation with cartilage degradation and increased CORO1A. scale bar:

200 μm.

A


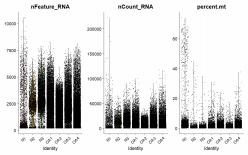


C


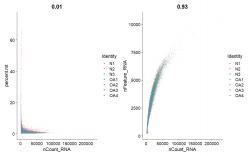


B


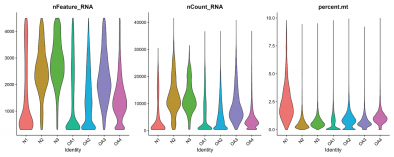


D


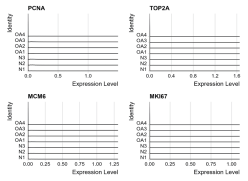


**Figure** **S3.** Quality control metrics for single-cell RNA sequencing data. **(A)** Integrated violin and scatter plots visualizing the distribution and relationship between three key quality control parameters before ﬁltering: total UMI counts (ncount RNA), number of detected genes (nFeature RNA), and mitochondrial gene percentage (percent.mt). Each point represents an individual cell. **(B)** violin plots displaying the distribution of the same three parameters after quality control ﬁltering. The applied ﬁltering criteria (nFeature RNA: 300-4500; percent.mt < 20%) effectively removed low-quality cells and potential multiplets, resulting in a more homogeneous cell population for downstream analysis. **(c)**scatter plots showing the relationship between total UMI counts (ncount RNA) and mitochondrial gene percentage (percent.mt, left panel) or number of detected genes (nFeature RNA, right panel) per cell. Most cells exhibit low mitochondrial content (<10%), indicating good cell viability, and display a positive correlation between sequencing depth and gene detection, reﬁecting expected biological variation. cells outside the main distribution in the left panel (high mitochondrial percentage) and right panel (low or extremely high gene counts) were ﬁltered out prior to downstream analysis. **(D)** Expression distribution of canonical proliferation markers across samples. Ridge plot showing the expression of four canonical proliferation markers (PcNA, TOP2A, McM6, MKI67) across different samples. The y-axis represents sample groups, and the x-axis represents normalized gene expression values. These markers exhibited consistently low expression levels in chondrocytes across all samples, which is in line with the terminally differentiated and lowly proliferative nature of cartilage tissue.
